# Comparative Analysis of Induced *vs.* Spontaneous Models of Autoimmune Uveitis Targeting the Interphotoreceptor Retinoid Binding Protein

**DOI:** 10.1371/journal.pone.0072161

**Published:** 2013-08-28

**Authors:** Jun Chen, Haohua Qian, Reiko Horai, Chi-Chao Chan, Yishay Falick, Rachel R. Caspi

**Affiliations:** 1 Immunoregulation Section, Laboratory of Immunology, National Eye Institute, National Institutes of Health, Bethesda, Maryland, United States of America; 2 Visual Function Core, National Eye Institute, National Institutes of Health, Bethesda, Maryland, United States of America; 3 Immunopathology Section, Laboratory of Immunology, National Eye Institute, National Institutes of Health, Bethesda, Maryland, United States of America; Oregon Health & Science University, United States of America

## Abstract

Animal models of autoimmunity to the retina mimic specific features of human uveitis, but no model by itself reproduces the full spectrum of human disease. We compared three mouse models of uveitis that target the interphotoreceptor retinoid binding protein (IRBP): (i) the “classical” model of experimental autoimmune uveitis (EAU) induced by immunization with IRBP; (ii) spontaneous uveitis in IRBP T cell receptor transgenic mice (R161H) and (iii) spontaneous uveitis in Autoimmune Regulator (AIRE)^−/−^ mice. Disease course and severity, pathology and changes in visual function were studied using fundus imaging and histological examinations, optical coherence tomography and electroretinography. All models were on the B10.RIII background. Unlike previously reported, IRBP-induced EAU in B10.RIII mice exhibited two distinct patterns of disease depending on clinical scores developed after onset: severe monophasic with extensive destruction of the retina and rapid loss of visual signal, or lower grade with a prolonged chronic phase culminating after several months in retinal degeneration and loss of vision. R161H and AIRE^−/−^ mice spontaneously developed chronic progressive inflammation; visual function declined gradually as retinal degeneration developed. Spontaneous uveitis in R161H mice was characterized by persistent cellular infiltrates and lymphoid aggregation, whereas AIRE^−/−^ mice characteristically developed multi-focal infiltrates and severe choroidal inflammation. These data demonstrate variability and unique distinguishing features in the different models of uveitis, suggesting that each one can represent distinct aspects of uveitis in humans.

## Introduction

Non-infectious uveitis involves a range of clinical pathologies including iritis, cyclitis, choroiditis, retinitis (including retinal vasculitis), and uveoretinitis, and is estimated to underlie 10–15% of blindness in the Western world [1], [2]. The etiology and pathogenesis are not fully understood and autoimmunity, which may in some cases also involve an autoinflammatory component, is believed to be involved [2], [3]. Due to practical and ethical limitations of human studies, the animal model of experimental autoimmune uveitis/uveoretinitis (EAU) [4], [5] has been used to study the basic mechanisms of disease. EAU can be induced in mice [6] and in rats [7]–[9] by active immunization with retinal antigens that are recognized by lymphocytes of uveitis patients, such as interphotoreceptor retinoid-binding protein (IRBP) and retinal soluble antigen (S-Ag). The main features of EAU in animals are retinal and/or choroidal inflammation, retinal vasculitis, photoreceptor destruction and loss of visional function [10]. As such they reproduce many essential clinico-pathological features of human uveitis [11].

Much of our current understanding of the basic mechanisms involved in uveitis has been derived from this “classical” EAU model. A limitation of the model is its dependence on use of complete Freund's adjuvant (CFA). CFA contains heat-killed mycobacteria which cause a massive stimulation of the innate immune response, and thereby also affect the adaptive response, in a way that may not be physiological. Therefore, the data should be compared to adjuvant-free models. Our group recently established a spontaneous model of uveitis in IRBP T cell receptor (TCR) transgenic (R161H) mice ([12] and Horai et al., submitted). R161H mice express a transgenic TCR specific to IRBP residues 161-180 (IRBP161-180; amino acid sequence SGIPYIISYLHPGNTILHVD) and spontaneously develop ocular inflammation by 5–6 weeks of age.

In addition to R161H mice, spontaneous autoimmune uveitis develops in mice deficient in the AIRE (AutoImmune Regulator) gene [13]. AIRE controls expression in the thymus of tissue-specific antigens, including IRBP and S-Ag, thus culling the most highly uveitogenic T cells from the repertoire. AIRE^−/−^ mice develop a multi-organ autoimmune disease that resembles human autoimmune polyendocrinopathy-candidiasis-ectodermal dystrophy (APECED), which also includes uveoretinitis [13], [14]. It is of note that uveitis in AIRE^−/−^ mice targets the IRBP antigen, as mice deficient in IRBP fail to develop retinal disease [15].

The pathology and clinical characteristics of the “classical” IRBP-induced EAU has been well characterized in previous studies [4], [10], [16]. However, the uveitis in R161H and AIRE^−/−^ mice has not been well characterized. In this study, we applied a series of non-invasive methods including fundoscopy, Micron-II fundus imaging, Bioptigen Envisu R2200 ultra-high resolution optical coherence tomography (OCT) system as well as electroretinography (ERG) to monitor the development and progression of uveitis longitudinally in individual animals. Clinical and histopathological findings as well as functional change of the retina are compared in the three murine models of uveitis. Our findings demonstrate variability and unique distinguishing features in each of the three uveitis models. The results suggest that while no single model represents the full spectrum of clinical and pathological features of human uveitis, each one can reproduce particular aspects of the human disease.

## Materials and Methods

### Animals, immunization for EAU and Ethics statement

B10.RIII mice were purchased from the Jackson Laboratory (Bar Harbor, Maine). Induction of EAU in B10.RIII mice was by active immunization with 6–8 µg IRBP emulsified in CFA (Sigma, St. Louis, MO) as described [17]. IRBP TCR transgenic R161H mice on B10RIII background were generated by the NEI Genetic Engineering Core using a TCR constrict cloned in our laboratory from a uveitogenic T cell line [18]. AIRE^−/−^ mice on the C56BL/6 background (B6.129S2-*Aire^tm1.1Doi^*/J, Stock# 004743), originally developed by M. Anderson et al [19] were purchased from Jackson Laboratory (Bar Harbor, Maine), and were backcrossed onto B10.RIII background. Animal care and use were in compliance with the guidelines of the National Institutes of Health and with the ARVO Statement for the Use of Animals in Ophthalmic and Vision Research. The animal study protocol was approved by the Animal Care and Use Committee of the National Eye Institute (animal study protocol NEI-581). All efforts were made to minimize any pain and distress, or relieve it through anesthesia/analgesia.

### Clinical evaluations

For all clinical examinations (fundoscopy, fundus photography, ERG, OCT) systemic anesthesia was by intraperitoneal injection of ketamine-xylazine (77 mg/kg+4.6 mg/kg, respectively). Local ocular surface anesthesia was by 0.5% Alcaine drops. The pupils were dilated using 0.5% Tropicamide and 0.5% phenylephrine hydrochloride. Frequency of examination varied from every other day during the acute phase of EAU to once weekly for chronic phase of EAU and the spontaneous models. While we cannot exclude that repeated anesthesia might have some effect on the disease process, ketamine/xylazine has not been reported to affect immune responses. Furthermore, all strains were anesthetized similarly, so a comparison across strains should remain valid.

### Scoring of uveitis by fundoscopy

Eyes were examined for clinical signs of uveitis using a binocular fundus microscope with coaxial illumination. A drop of sterile physiological solution was placed on the cornea and a microscope coverslip served as a lens to equalize refraction. Eyes were examined for engorged blood vessels, constricted blood vessels (“cuffing”), white linear lesions, subretinal hemorrhages, and retinal detachment. Clinical uveitis was scored on a scale of 0–4, based on the number and type of retinal lesions, as described in detail elsewhere [20], [21].

### Digital fundus imaging

Mice were anesthetized and their pupils dilated as above. The fundus was imaged using the Micron II small animal retinal imaging AD camera (Phoenix Research Laboratories, INC).

### Spectral Domain Optical Coherence Tomography (SD-OCT) imaging

Mice were anesthetized and their pupils dilated as above. Artificial tears (Systane Ultra, Alcon) were used throughout the procedure to maintain corneal moisture and clarity. SD-OCT images were obtained using the Bioptigen Spectral Domain Ophthalmic Imaging System (SDOIS; Bioptigen Envisu R2200, North Carolina) using proprietary Bioptigen software. Single B scan and volume scans were obtained with images centered on the optic nerve head. Seventeen B10.RIII, sixteen R161H and twenty-one AIRE^−/−^ mice comprising two to three repeat experiments were included in the longitudinal study.

### Assessment of retinal thickness

Retinal thickness was measured manually from each averaged B-scan OCT image, approximately 1 optic disc diameter (∼50 µm) away from the both edges of the optic disc. Retinal thickness was defined as distance between inner limiting membrane and Bruch's membrane from the intensity peak of boundary corresponding to the interface of the vitreous and the ganglion cell layer (GCL) to the intensity peak corresponding to the interface between the retinal pigment epithelium (RPE) and choroid [22].

### Assessment of cellular infiltrates in the vitreous

Volume-scan of OCT images containing cellular infiltrates was captured in the vitreous, between the posterior capsule of the lens and the retinal ganglion cell layer (GCL). All images were processed digitally in the same way, using Photoshop to standardize background contrast levels. OCT signal intensity, which reflects intensity of cellular infiltrates in the vitreous, was measured and analyzed using ImageJ software (NIH).

### Electroretinography (ERG)

Retinal function was evaluated by recording of dark- and light-adapted ERG with the Espion E2 System (Diagnosys LLC). Mice were dark adapted overnight, and all procedures were performed under dim red light. Mice were anesthetized and their pupils dilated as described above. For the ERG recordings, electrodes were placed on the center of cornea. Reference and ground electrodes were attached to the mouth and placed subcutaneously in the posterior neck-back region respectively. The a-wave amplitude was measured from the baseline to the trough of the a-wave, and b-wave amplitude was measured from the trough of the a-wave to the peak of the b-wave. Representative ERG plots can be seen in Figure S1.

### Histology

Eyes were harvested at different time points after immunization, or at different ages after development of spontaneous uveitis, fixed in 10% formalin, embedded in methacrylate, sectioned through the pupillary-optic nerve plane and stained with hematoxylin-eosin. The severity of EAU was evaluated in a masked fashion on a scale of 0–4 using previously published criteria based on the number, type, and size of lesions [10]. Sixteen to twenty-one mice of each strain were included in the histological examination, and eyes of 2–3 mice were harvested at each time point.

### Statistical analysis

All data are expressed as mean ± SEM. Statistical analyses were performed using the Mann-Whitney U test. P≤0.05 was considered statistically significant.

## Results

### Disease severity in induced and spontaneous models of uveitis by fundus examination

**Figure 1:**
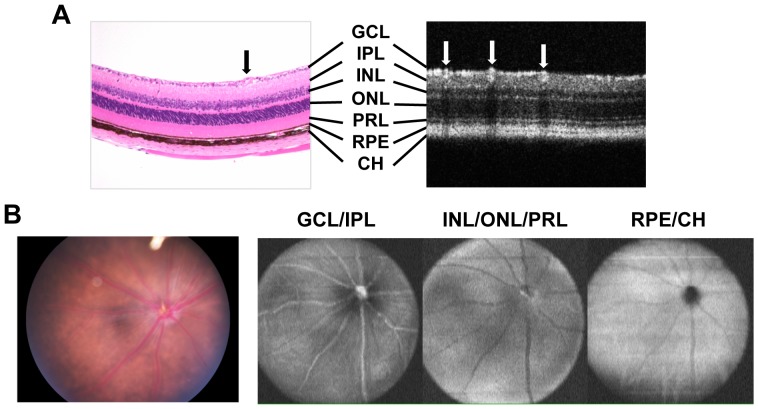
Comparison of retinal images obtained by fundus photography, OCT, and histology in B10.RIII mouse. **A,** Normal retinal layers in a healthy eye assessed by cross-sectional OCT in comparison with histology. Note the appearance of the ganglion cell layer (GCL), inner plexiform layer (IPL), inner nuclear layer (INL), outer nuclear layer (ONL), IS/OS of photoreceptor layer (PRL), retinal pigment epithelium (RPE) and choroid (CH) in the respective images. Blood vessels are indicated by arrows. **B,** Comparison of fundus appearance by Micron II imaging with OCT volume scan images in multiple retinal layers of normal eye.

**Figure 2:**
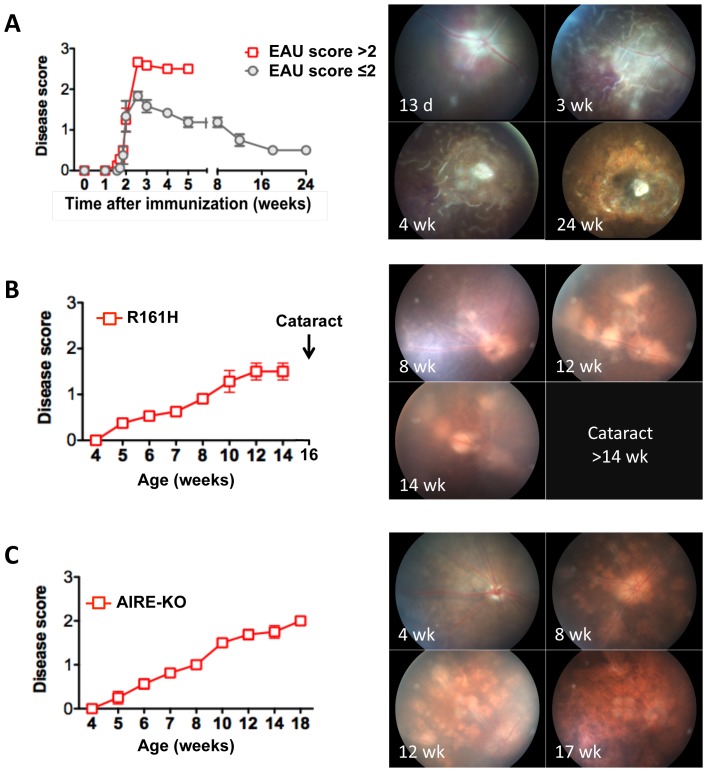
Comparison of disease severity and course in the induced and spontaneous uveitis models. EAU was induced in B10R.III mice by immunization with IRBP161-180 in adjuvant. Clinical scores at different stage of disease were evaluated using an adapted fundus microscope. **A,** EAU induced by immunization with IRBP in adjuvant; Fundus photographs shown is the chronic form of disease seen in 4/17 B10RIII mice. **B,** R161H uveitis (n = 16); **C,** AIRE^−/−^ uveitis (n = 21). Fundus photographs in the same eye were taken using a Micro-II fundus imaging camera. Representative fundus images are shown. Data are presented as mean ± SEM of 17 mice from two individual experiments (A) and 16–21 mice from two to three individual experiments (B, C).

The appearance of healthy ocular tissues visualized by the methodologies used in this study is shown in Figure 1 and serves as comparison for all subsequent Figures. Disease severity and course were evaluated and compared between three models of uveitis, the “classical” immunization-induced EAU model, spontaneous uveitis developing in R161H IRBP TCR transgenic mice, and spontaneous uveitis developing in AIRE^−/−^ mice (Figure 2). A common platform for comparison is present because all 3 models were on the B10.RIII background and in all 3 models IRBP is the target antigen. In the immunization-induced model, EAU onset occurred 11–13 days post immunization (p.i.) and peaked on day 14. In contrast to the generally held belief that EAU in B10.RIII mice is strictly an acute monophasic inflammatory disease, two distinct patterns of EAU progression were observed: monophasic and chronic. The progression pattern of individual animals depended on the severity of the initial acute phase of retinal inflammation (depicted in Figure 2A). (i) In the monophasic EAU pattern, mice that developed disease with clinical scores of EAU greater than 2 in the acute phase of inflammation during weeks 2–3 p.i., manifested a rapid resolution of active inflammation (consisting of perivascular exudates, subretinal hemorrhage and retinal folds, etc.) after week 3, as well as thinning of the retina. Active inflammation disappeared around 4–5 weeks p.i., and was followed rapidly by retinal atrophy that marked the end of clinical scoring for EAU. (ii) In the chronic EAU pattern, following the initial phase of acute retinal inflammation 2–3 weeks p.i., mice that had developed clinical disease scores of 2 or less during the acute phase exhibited chronic ocular inflammation manifested as persistent white granulomatous like lesions and perivascular cuffing that continued for 5–6 months until retinal degeneration and atrophy occurred. In the current study, using 6–8 µg IRBP161-180/mouse immunization dose, about ¾ of mice developed monophasic disease, and ¼ developed chronic disease.

In contrast to the induced model, R161H mice spontaneously developed chronic-progressive ocular inflammation. R161H mice showed an early onset of disease at 5–6 weeks of age. Retinal inflammation was characterized by persistent and aggressive infiltrates and large white perivascular lesions in the retina that had a bead-like appearance. Disease reached a peak at 10 weeks of age at which time the mice gradually started to develop secondary cataracts, essentially precluding further follow up by fundus examination beyond week 14 (Figure 2B).

Chronic-progressive ocular inflammation was also observed in AIRE^−/−^ mice, which spontaneously developed uveitis at 5–6 weeks of age, similarly to R161H. However, unlike the appearance of the lesions in R161H mice, AIRE^−/−^ mice developed a progressive multifocal retinal and choroidal inflammation. The foci progressively coalesced and led to retinal destruction and atrophy at 10–14 weeks of age (Figure 2C). No cataracts were formed during the course of study in AIRE^−/−^ mice, possibly due to less aggressive ocular inflammation in this model.

### Disease activity measured by retinal thickness in induced and spontaneous uveitis models

**Figure 3:**
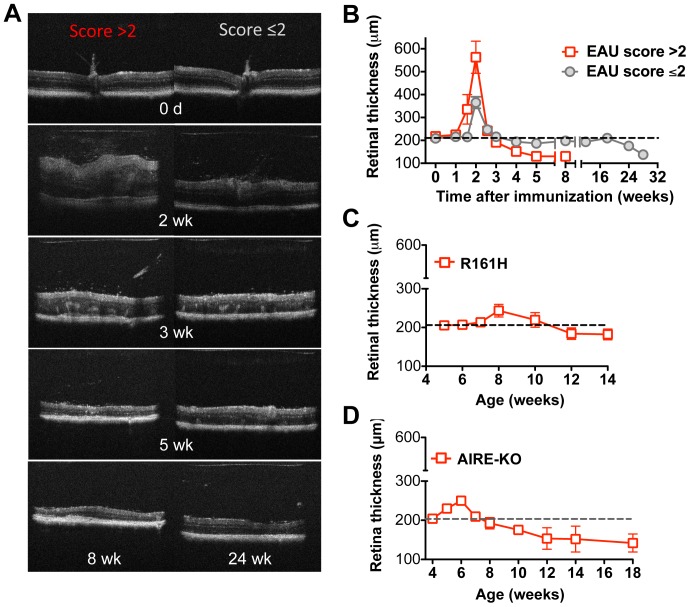
Changes in retinal thickness by OCT imaging in induced and spontaneous uveitis models. **A–B,** EAU was induced in B10.RIII mice by active immunization with IRBP161-180 in adjuvant. B-scan OCT of the retina was evaluated at the indicated time points using a Bioptigen SD-OCT imaging system. Shown are representative examples of retinal thickness at different stages of monophasic and chronic forms of disease (A). Retinal thickness was measured and averaged from OCT images of the retina (B). **C–D,** Retinal thickness of R161H mice and AIRE^−/−^ mice was evaluated at different ages using a Bioptigen SD-OCT imaging system. Data are presented as mean ± SEM of 17 mice from two individual experiments (A–B) and 16–21 mice from two to three individual experiments (C–D).

An additional parameter that can give a measure of disease activity is retinal thickness, as measured from B-scan OCT imaging [22]. By this criterion as well, two patterns of disease progression were observed in the immunization-induced model of EAU, depending on the severity of the initial acute phase of disease (Figures 3A and 3B). (i) In the acute pattern, increase in retinal thickness could clearly be detected already at 11–12 days p.i., reaching a peak of 2–3 fold increase in retinal thickness at 2 weeks, which corresponded to peak of clinical inflammation as detected by fundus examination. Characteristically, mice that had developed disease with clinical fundoscopy scores higher than 2 manifested a sharp decline of retinal thickness under baseline starting at 3 weeks p.i. Active inflammation ended around 4–5 weeks followed by what appeared to be retinal atrophy with retinal thickness of only about half of the baseline. (ii) In the chronic pattern of EAU, mice that initially developed clinical disease scores of 2 or less, went on to develop a chronic pattern of retinal inflammation. No retinal thickness changes were detected at 11–12 d p.i. and retinal thickness peaked at <2-fold on 2 weeks p.i. Retinal thickness quickly returned to nearly normal baseline and remained close to this level for several months. After 5–6 months of chronic disease, thinning of the retina (retinal degeneration) occurred. In both patterns of induced EAU, the increase of retinal thickness corresponded to disease activity as observed by clinical EAU scoring (Figure 2A) [22].

In contrast to the induced EAU model, the spontaneous models of uveitis seen in R161H and AIRE^−/−^ mice showed relatively slow and progressive changes with a mild increase in retinal thickness during the peak of inflammation around 6–8 weeks of age. Beyond 10–12 weeks, thinning of the retina, indicating degeneration/atrophy occurred in both spontaneous models (Figure 3C and 3D), but in R161H mice, development of cataracts at 14 weeks of age precluded OCT examination beyond that point. In AIRE^−/−^ mice, on the other hand, progressive thinning of the retina was documented, which reduced retinal thickness at the late phase of disease.

### Quantitation and kinetics of vitreous cellular infiltrates during induced and spontaneous uveitis by OCT imaging

**Figure 4:**
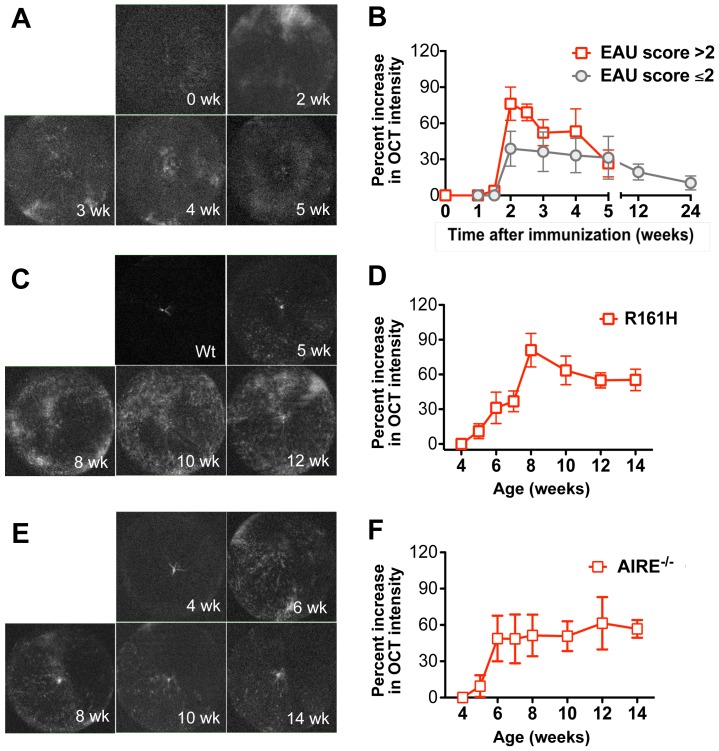
Semi-quantitative evaluation of cellular infiltrates in the vitreous of mice with induced and spontaneous uveitis. **A,** Longitudinal imaging of volume scan OCT was performed during the course of EAU in the vitreous of the same eyes as in Figure 2. (A,B) IRBP induced EAU (n = 8); (C,D) R161H (n = 8); (E,F) AIRE^−/−^ (n = 6). **B,** Semi-quantitative evaluation of cellular infiltrates over time. Volume-scan of OCT images was captured in the vitreous (Materials and Methods). All images were digitally processed in the same way using Photoshop to equalize background contrast levels. Signal intensity of OCT volume scan was then measured and analyzed using ImageJ analysis. Data are presented as mean ± SEM of percent increase of OCT intensity to normal or WT mice.

Vitreous haze and aqueous flare are useful clinical parameters to quantitate inflammation in the patient [23]. In the 3 mouse models, we monitored the kinetics of cellular infiltration into the vitreous with volume-scan OCT images shown in Figure 4 (A,C,E), and semi-quantitatively evaluated the cellular infiltration by processing and measuring OCT signal intensity using ImageJ analysis shown in Figure 4 (B,D,F).

In WT mice immunized for EAU that developed monophasic disease, massive cellular infiltrates were observed in the vitreous during the acute phase 2–4 weeks after immunization, which dissipated rapidly starting 4–5 weeks post immunization. In contrast, mice that developed the chronic form of EAU exhibited a more moderate infiltrate in the vitreous during the acute phase. The cell infiltrate remained at a moderate level until 12 weeks after immunization, and receded thereafter.

In R161H mice, cellular infiltrates became apparent in the vitreous as early as 4–5 weeks of age, marking disease onset. The cellular infiltration increased rapidly, reaching a peak at 8 weeks of age, and then remained for 2–3 months until onset of secondary cataracts occurred at 14 weeks, which precluded further OCT recordings. Similarly, vitreous infiltration became apparent in AIRE^−/−^ mice around 5–6 weeks of age. The level of cellular infiltration was relatively modest compared to that in R161H mice, persisting for 3–4 months until retinal degeneration occurred and further observation was discontinued.

### Comparative histopathology of induced and spontaneous uveitis models

**Figure 5:**
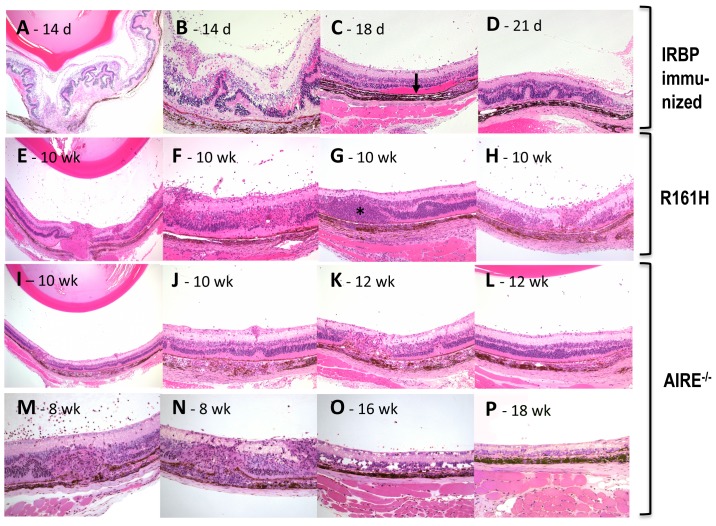
Histopathology of induced and spontaneous uveitis models. **A–D,** Mice were immunized with IRBP in adjuvant and eyes were collected at the indicated days after immunization. Note severe ocular inflammation including vitritis, retinal swelling and destruction, retinal folds and infiltrates, subretinal hemorrhage, and choroidal inflammation at the peak of inflammation on day 14 p.i. (A–B, low and high magnification). During the acute phase of EAU, 18–21 days after immunization, eye histology showed well developed retinal lesions and infiltrating cells in the choroid, vitreous, as well as subretinal hemorrhage (arrow) (B and C). Retinal folds were seen in mice that developed the chronic form of EAU (D). **E–H,** Histology of R161H mice at different ages. Note cellular infiltrates and exudates in the vitreous and in the retina (E–F, low and high magnification), lymphoid aggregation in the retina (G, asterisk), photoreceptor layer destruction (H) and choroidal inflammation (G–H). **I–P,** Histology of AIRE^−/−^ mice at different ages. Note severe choroiditis (I–J, low magnification; N, high magnification), granuloma-like lesions in the retina (K, low magnification; M–N, high magnification), photoreceptor layer destruction and retinal degeneration (O–P, high magnification). Fourteen B10RIII mice with EAU (10 for monophasic form, 4 for chronic form), 12 R161H and 13 AIRE^−/−^ mice were included in the histological examination. Eyes of 2–3 mice were harvested at each time point.

We next compared the histopathology of the eyes in the induced and spontaneous models of uveitis (Figure 5). At the peak of IRBP-induced monophasic EAU, on day 14 after immunization (Figure 5A–B), ocular histology showed severe subacute inflammatory cellular infiltration into the vitreous and choroid, along with iridocyclitis, retinal edema, folds and infiltrates. Shortly after that retinal edema diminished, but discrete retinitis, vitreal and subretinal hemorrhage, retinal folds, as well as choroiditis were prominent on days 18–21 after immunization (Figure 5C). Later the retina became diffusely degenerative and atrophic (see Figure 3A). In mice that developed the chronic form of EAU retinal folds were also frequently observed 18–21 days after immunization (Figure 5D) but atrophy at that point was focal at most, and did not set in until 6–7 months.

Chronic and progressive ocular inflammation was apparent in R161H mice (E–H), which was characterized by monocytic cellular infiltration in the vitreous and retina. Characteristic focal lymphoid aggregates were often found in the retina, accompanied by photoreceptor destruction. Chronic-progressive ocular inflammation was also observed in AIRE^−/−^ mice (I–P). Ocular histology of these mice was characterized by moderate retinitis and severe choroiditis, along with prominent retinal atrophy at later stages of disease.

### OCT imaging of retinal lesions in induced and spontaneous uveitis models

**Figure 6:**
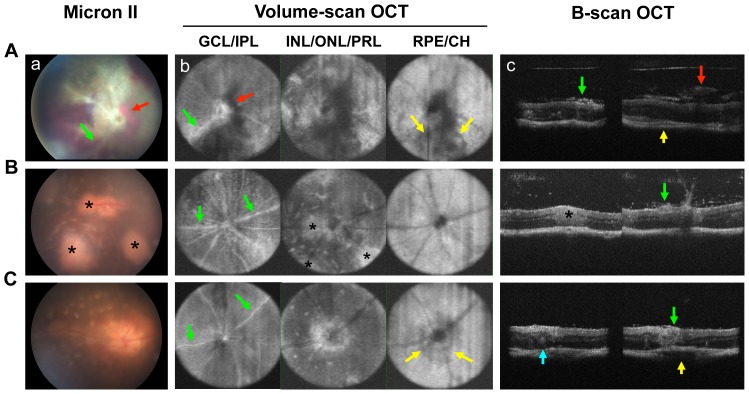
Distinguishing features of retinal inflammation in induced and spontaneous uveitis models. Retinal lesions were visualized using Micron-II fundus imaging and Bioptigen SD-OCT imaging systems in mice with induced EAU (A), R161H (B) and AIRE^−/−^ (C) mice. Shown are: (a) fundus images; (b) three volume-scan OCT images representing the ganglion cell and inner plexiform layer (GCL/IPL), the inner/outer nuclear and inner/outer segments of the photoreceptors layer (INL/ONL/PRL), and the retinal pigment epithelium/choroid layer (RPE/CH); (c) B-scan OCT. All images are of the same eye. Note engorged blood vessels and peri-vascular exudates (green arrow) in GCL/IPL, vitreal and subretinal hemorrhages (red arrow, dark area) visible in all retinal layers (and corresponding to the same lesions in the fundus image and in OCT B-scan) and inflammation (yellow arrow) in RPE/CH.

We next examined the retinal pathology using non-invasive fundus imaging *vs.* SD-OCT imaging systems. Figure 6 shows volume-scan OCT image slices of different retinal layers compared to digital fundus photographs in induced and spontaneous uveitis and B-scan OCT images. All observations were performed in parallel on the same eyes.

As we reported previously [22], OCT imaging was able to distinguish retinal pathology in different retinal layers *in vivo*. In mice developing severe monophasic EAU 18 days after immunization, OCT was able to partially overcome the difficulty of visualization of the retinal layers due to hazy media resulting from extensive inflammation in the anterior and posterior segments of the eye. In keeping with the fundus images of the same eye, horizontal-sections of OCT volume scan images exhibited engorged blood vessels and peri-vascular exudates (green arrow) in ganglion cell and inner plexiform layers (Figure 6A). Severe vitreal and subretinal hemorrhages (red arrow, dark area) in ganglion cell/inner plexiform layer as well as in inner/outer nuclear and IS/OS of photoreceptor layer recapitulated the pattern seen in the fundus image. Inflammation (yellow arrow) was also noted in RPE and choroid layers. Cross-sections of OCT B-scan images also resolved the vitreal hemorrhage (red arrow) and cellular infiltrates (green arrow) observed by fundus imaging in the retina, and choroiditis (yellow arrow).

The spontaneous uveitis in R161H mice was characterized by perivascular infiltrates in the retina (Figure 6B). In correlation to the fundus images, B-scan OCT imaging recorded multifocal lesions of cellular aggregation (asterisk) penetrating through inner/outer nuclear and IS/OS of photoreceptor layers of the retina. Retinal lesions, including marked cellular infiltration in the vitreous and perivascular exudates in ganglion cell layer (green arrow) were also resolved by B-scan OCT images.

Retinal inflammation in AIRE^−/−^ mice was characterized by perivascular cellular infiltration penetrating the retinal layers (green arrow), numerous localized retinal folds observed in inner/outer nuclear and IS/OS of photoreceptor layers (blue arrow), swelling of optical nerve and severe choroiditis (yellow arrow, Figure 6C).

### Changes in visual function in the induced and spontaneous models of uveitis

**Figure 7:**
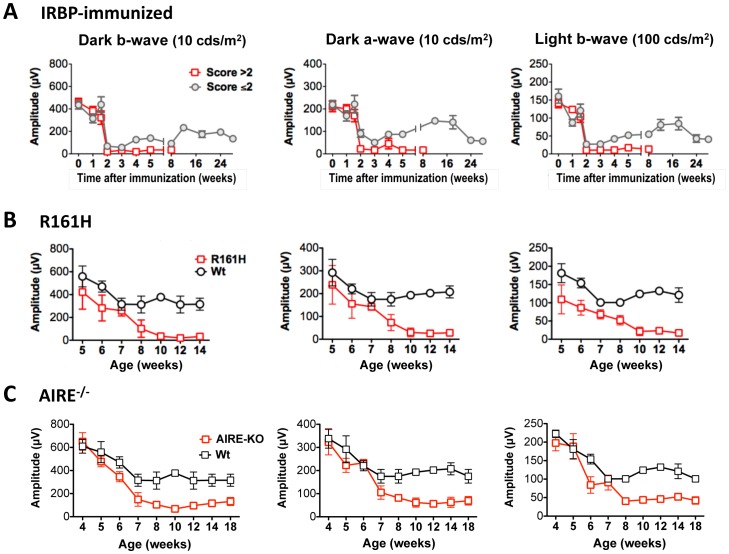
Kinetics of dark- and light-adapted ERG response in induced and spontaneous uveitis models. Mice that developed induced and spontaneous EAU were monitored and followed up at the indicative time points by ERG. Amplitude of dark- and light-adapted ERGs was recorded and analyzed in mice developed IRBP-induced EAU (A) and in R161H (B) and AIRE^−/−^ (C) mice that developed spontaneous uveitis. Data represent the mean ± SEM of 17 mice from two individual experiments (A) and 16–21 mice from two to three individual experiments (B, C).

Visual function of mice with the different forms of uveitis was examined using an ERG recording system in the same mice whose retinal changes were measured by OCT, fundus imaging and histology, and were depicted in previous Figures. We followed both scotopic (dark-adapted) and photopic (light-adapted) ERG responses during the course of the disease (Figure 7). Although uveoretinitis affected rod (scotopic) and cone (photopic) signals in a similar fashion, the pattern of visual loss differed markedly among the three models, and differed between the monophasic and chronic forms of the induced model.

In the induced EAU model, two characteristic patterns of ERG responses could be distinguished that corresponded to the monophasic and chronic courses of disease in mice which developed scores >2 or ≤2, respectively, during the initial acute phase of disease (Figure 7A). Following the onset of disease on day 11 after immunization, mice destined to develop monophasic disease as well as mice destined to develop chronic disease, experienced a detectable reduction in both b-wave and a-wave amplitudes measured in both dark- and light-adapted conditions. By day 14, when inflammation and retinal edema reached its peak (see Fig. 3A), the b- and a-wave amplitudes in response to both dark and light adaptations dropped precipitously by 90% and remained flat during the acute phase of disease. However, whereas in the monophasic group ERGs continued to remain flat when inflammation resolved after day 21, in the chronic group resolution of acute inflammation and transition to the chronic phase was followed by partial recovery of visual function with ERGs returning to 50–65% of normal amplitude. The partial return of visual function lasted for several months, despite the chronic low-grade retinal inflammation, and was followed by a detectable decline when retinal thickness dropped under baseline, indicating retinal degeneration, 6–7 months after immunization.

ERG recording in R161H and AIRE^−/−^ mice, as well as their wild type (WT) littermates, was initiated at 4–5 weeks of age, in keeping with the early onset of spontaneous disease in these models. We noted that, as part of what appeared to be normal postnatal development of the retina, ERG amplitudes measured on WT mice exhibited a downward trend through puberty, to plateau at about 7 weeks of age (Figure 7B). R161H mice, in which clinical uveitis can manifest as early as 4 weeks of age, consistently exhibited ERG responses below the corresponding WT values already at week 4. After week 7, when their chronic retinal inflammation started to peak as judged by fundus examination and retinal thickness, a rapid decline of ERG values was seen, which continued progressively and finally plateaued at near zero levels after week 10, when reduction of retinal thickness, a hallmark of retinal degeneration, became apparent (Figure 7B). Although AIRE^−/−^ mice initially exhibited ERGs similar to WT controls (up to 5 weeks) the values dipped below WT thereafter. As in R161H mice, AIRE^−/−^ ERG values dropped rapidly after week 7 to plateau at a low level after week 10, when appreciable thinning of the retina occurred, reflecting a permanent deficit in visual function.

## Discussion

Experimental animal models are necessary preclinical tools for studying disease pathogenesis and translational immunotherapies. In the present study, we characterized and compared three distinct models of uveitis: the “classical” EAU model induced by immunization and two spontaneous models in IRBP TCR transgenic R161H mice and AIRE^−/−^ mice. To document the disease course, severity, pathological findings and functional changes of the retina in the different models as comprehensively as possible, we used fundoscopic and histological examinations, fundus imaging and SD-OCT imaging systems, as well as ERG recordings. The salient features of the induced vs. spontaneous models of uveitis are summarized in Table 1.

**Table 1:** Summary of morphologic and functional changes in the different models of uveitis.

Model	Onset and progression of ocular inflammation
***IRBP-induced EAU (active immunization)***	
B10.RIII	• Monophasic & chronic forms of posterior uveitis; transient anterior uveitis
	• Onset of disease 11d after uveitogenic challenge
	• Diffuse retinal lesions; extensive exudates & infiltrates; destruction of photoreceptor cell layer
	• A proportion of the mice (lower initial scores) develop chronic form of disease
	• Decline of ERG at peak of inflammation (monophasic) & retinal atrophy phase (chronic) but partial recovery of retinal function for several months in chronic form
***Spontaneous uveitis***	
R161H (Tg)	• Chronic form of posterior uveitis
	• Spontaneous uveitis starts at early age
	• Minimal anterior chamber involvement
	• Focal retinal lesions; persistent cellular infiltrates & aggregates
	• Decline of ERG during retinal atrophy phase
AIRE^−/−^	• Chronic form of posterior uveitis
	• Spontaneous uveitis starts at early age
	• Lack of anterior chamber involvement
	• Small focal retinal & choroidal lesions; less cellular infiltrates
	• Decline of ERG during retinal atrophy phase

The kinetics of disease onset and progress were visualized by fundus photography, OCT imaging, histology and ERG. The morphologic and functional changes in three models of uveitis are summarized based upon observation.

A notable observation was the prominent differences in the clinical and histological manifestations and progression of uveitis in the three models, especially in view of the fact that all are on the same B10.RIII background and all are driven by a response to IRBP [13], [24], [25]. We believe that this is in part attributable to the differences in the disease triggering mechanisms, i.e., active immunization in CFA with its associated activation of innate immune arm *vs.* spontaneous disease in the context of enhanced precursor frequency of uveitogenic T cells, which could translate to differences in cytokine/chemokine profiles. We previously reported that CFA promotes a Th17-dominant response and that IL-17 is necessary for induction and for progression of uveitis induced in the context of CFA [26]. In contrast, preliminary data in R161H mice implicate IFN-γ as an important cytokine (Horai, Chen et al., in preparation). The dominant effector response in AIRE^−/−^ mice has been reported to be Th1 [15], but not all tissue pathologies appeared Th1 dependent, and Th1 dependence of AIRE^−/−^ uveitis had not been specifically examined.

It is a matter of debate which model best represents human disease. Since human uveitis is heterogeneous, the answer may well be that all are relevant. Induced EAU with its rapid onset, explosive development and diffuse pathology appeared to approximate acute and subacute forms of human uveitis better than the spontaneous uveitis models. In contrast, the spontaneous models with their relentless chronic-progressive course, but a more focal type of pathology, could more faithfully represent chronic types of human uveitis. An interesting finding that had not been reported previously, is that the induced model in B10.RIII mice can take on a chronic course if mice develop relatively lower scores in the initial acute phase of EAU. Such mice went on to develop a chronic-progressive disease, with significant recovery of visual function for several months, although after several months of low-grade chronic inflammation they ultimately succumbed to retinal degeneration and permanent visual loss. While a relapsing EAU course had been described in B10.A mice [27] and a chronic course had been documented in C57BL/6 mice [28], [29], the EAU model in the B10.RIII strain had until now been considered to only manifest an acute-monophasic pattern [21], [30]. The reason that the chronic form disease had not been previously appreciated could be the high susceptibility of the B10.RIII strain to EAU (some laboratories even use pertussis toxin to further enhance disease scores) and because few if any studies have followed these mice over the long term. During the acute phase, the two forms of disease appear clinically similar and unless the mice had been immunized with a relatively low dose regimen and followed well after the acute phase resolves, the chronic form would be missed.

In addition to the extensive retinal inflammation, mice immunized for EAU with IRBP/CFA developed severe but transient anterior uveitis during the peak of acute disease, manifesting as an opaque anterior chamber with aqueous proteinaceous exudates and cellular infiltrates in the iris and ciliary body. In contrast, anterior uveitis was minimal or absent in the spontaneous models. Microbial triggers are suspected contributors to several forms of immune-mediated, noninfectious forms of anterior uveitis including that associated with ankylosing spondylitis, sarcoidosis, Behçet's disease and inflammatory bowel disease [31]. As well, both rats and mice develop a short-term and self-limited anterior uveitis after a systemic or local injection of microbial products such as lipopolysaccharide, muramyl dipeptide, and lipoteichoic acid [32]–[37]. Although the mechanisms are not fully understood, it is possible that the bacterial components present in the complete Freund's adjuvant facilitate anterior chamber inflammation, in addition to the posterior inflammation.

It is apparent from the present study that combining several types of methodologies can yield information about a model that could not be obtained by one methodology alone. The clinical fundus scoring is based on the currently accepted EAU scale, which was based on the acute phase of the immunization-induced disease [20], [21]. Some parameters, including vitreous infiltrates, anterior chamber inflammation (based on clinical findings of aqueous cells and flare, posterior synechiae and histological findings of inflammatory cells and protein exudate in the anterior chamber, iris, and ciliary body), cataract formation and retinal degeneration, are not accommodated by this scale. Our ERG data show that retinal function is rapidly lost when acute inflammation sets in, but may recover for long periods of time despite persistence of low-grade inflammation during the chronic phase of IRBP-induced EAU. In contrast, in the AIRE^−/−^ and R161H mice that developed chronic, but focal, lesions, visual function declined gradually and the pattern of ERG loss could not distinguish between an inflammatory phase and a degenerative phase. However, fundus examination and measurement of retinal thickness by OCT were able to resolve differences in the appearance and progression of uveitis in these two models. Inflammation in R161H mice was characterized by minimal anterior chamber involvement (primarily limbitis) but prominent, persistent and aggressive cellular infiltrates and lymphoid aggregates in the retina that led to secondary cataract around 14 weeks of age, precluded further fundoscopic examination, but ERG and OCT as well as histological evaluation continued to be useful in documenting development of their disease. AIRE^−/−^ mice lacked anterior uveitis and the characteristic feature of their disease was a relatively low-grade but multi-focal retinal inflammation along with severe choroiditis, that subsequently led to retinal degeneration. As an aside, we noted that ERG amplitudes measured on healthy WT mice exhibited a downward trend until onset of puberty at about 7 weeks of age, appearing to reflect normal postnatal development of the retina. It is unknown whether this is a characteristic of the species, or something restricted to this strain.

In summary, the present study demonstrates the variability, and uncovers the unique distinguishing features, in induced and spontaneous models of uveitis. In addition to the differences in the course of disease, particularly prominent was the anterior chamber involvement in EAU, which was minimal in R161H and absent in AIRE^−/−^ mice. Between R161H and AIRE^−/−^ mice, the main differences were focal retinal lymphoid aggregates in R161H vs. prominent choroidal involvement in AIRE^−/−^ mice. R161H pathology thus appears more localized to the retina, whereas AIRE^−/−^ pathology targets the choroid as well. Our findings underscore that although no one single animal model represents the full spectrum of clinical and pathological features of human uveitis, each one can reflect particular aspects of human disease.

## Supporting Information

Figure S1 **Representative examples of dark- and light-adapted ERG plots in induced and spontaneous uveitis models.** Mice that developed induced and spontaneous uveitis were examined at the indicated time points by ERG. Amplitude of dark- (A) and light-adapted (B) ERGs was recorded and analyzed in mice that developed IRBP-induced EAU and in R161H and AIRE^−/−^ mice that developed spontaneous uveitis. Data are representative of ERG plots from 16–21 mice from two to three individual experiments.
(TIF)
