# Supplementary figures and images for: Comparative Analysis of Induced vs. Spontaneous Models of Autoimmune Uveitis Targeting the Interphotoreceptor Retinoid Binding Protein
Source: PLoS One. 2013 Aug 28;8(8):e72161. doi: 10.1371/journal.pone.0072161 (PMC3756070; doi:10.1371/journal.pone.0072161)

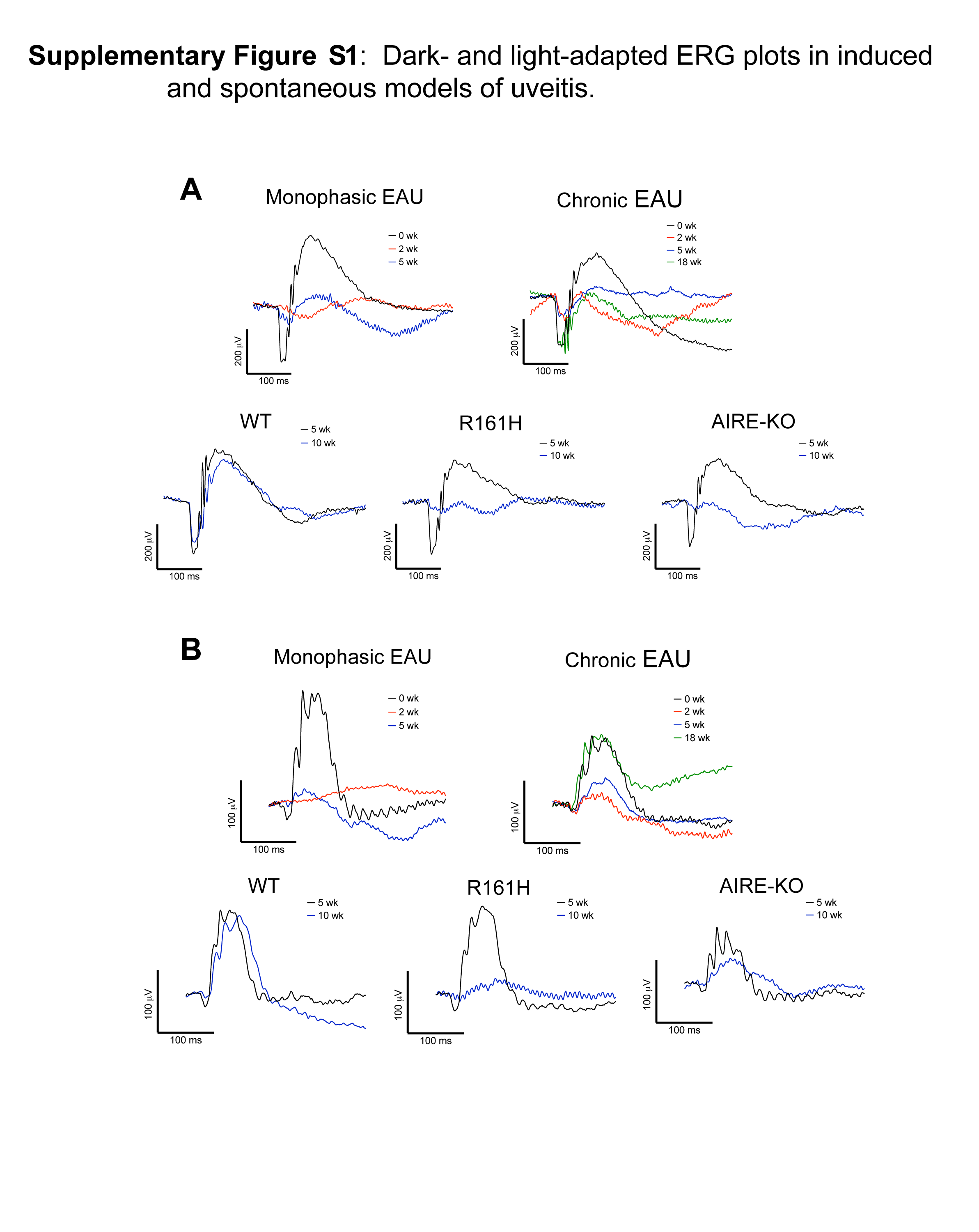

Supplement: Figure S1 — Representative examples of dark- and light-adapted ERG plots in induced and spontaneous uveitis models. Mice that developed induced and spontaneous uveitis were examined at the indicated time points by ERG. Amplitude of dark- (A) and light-adapted (B) ERGs was recorded and analyzed in mice that developed IRBP-induced EAU and in R161H and AIRE−/− mice that developed spontaneous uveitis. Data are representative of ERG plots from 16–21 mice from two to three individual experiments. (TIF) [file pone.0072161.s001.tif]
